# Non-proteolytic calpain-6 interacts with VEGFA and promotes angiogenesis by increasing VEGF secretion

**DOI:** 10.1038/s41598-019-52364-6

**Published:** 2019-10-31

**Authors:** Mijung Oh, Seung Bae Rho, Chaeyeun Son, Kyoungsook Park, Sang Yong Song

**Affiliations:** 1 Department of Pathology and Translational Genomics, Samsung Medical Center, Sungkyunkwan University School of Medicine, Seoul, 06351 Republic of Korea; 2 Division of Translational Science, National Cancer Center, Ilsan-ro 323, Ilsan-gu, Goyang, 10408 Republic of Korea; 3 Medical Research Center, Sungkyunkwan University School of Medicine, Suwon, 16419 Republic of Korea

**Keywords:** Tumour angiogenesis, Tumour angiogenesis

## Abstract

Angiogenesis is involved in both normal physiological and pathological conditions. Vascular endothelial growth factor (VEGF) is a major factor for promoting angiogenesis. The current anti-VEGF therapies have limited efficacy and significant adverse effects. To find novel targets of VEGFA for angiogenesis inhibition, we performed yeast two-hybrid screening and identified calpain-6 as a novel VEGFA-interaction partner and confirmed the endogenous VEGFA–calpain-6 interaction in mammalian placenta. A domain mapping study revealed that the Gly321–Asp500 domain in calpain-6 is required for the interaction with the C-terminus of the VEGFA protein. The functional significance of the VEGFA–calpain-6 interaction was explored by assessing its effect on angiogenesis *in vitro*. Whereas forced overexpression of calpain-6 increased the secretion of the VEGF protein and tube formation, knockdown of calpain-6 expression abrogated the calpain-6-mediated VEGF secretion and tube formation in HUVECs. Consistent with the domain mapping result, overexpressing calpain-6 without the VEGFA-interacting domain III (Gly321–Asp500) failed to increase the secretion of VEGF protein. Our results identify calpain-6, an unconventional non-proteolytic calpain, as a novel VEGFA-interacting protein and demonstrate that their interaction is necessary to enhance VEGF secretion. Thus, calpain-6 might be a potential molecular target for angiogenesis inhibition in many diseases.

## Introduction

Angiogenesis is a complex process that is stimulated by a variety of growth factors, including vascular endothelial growth factor (VEGF). Angiogenesis is involved in various normal physiological conditions, such as embryogenesis and placental growth, as well as pathological conditions, including tumor growth, diabetic retinopathy, rheumatoid arthritis, and ischemic diseases^1^. Due to the well-established role of VEGF in promoting tumor angiogenesis and the pathogenesis of human cancers, most of the angiogenesis inhibitors that are used clinically for cancer treatment target the VEGF-VEGF receptor (VEGFR) pathway^2–4^. Although anti-VEGF/VEGFR therapies have demonstrated potent inhibition of angiogenesis and tumor growth in preclinical models^5,6^, accumulating clinical data have shown that VEGF-targeted therapies do not benefit all cancer patients and can lead to serious adverse side effects^7–9^. Thus, novel targets for angiogenesis inhibition need to be identified to establish new anti-angiogenesis strategies and improve current anti-VEGF therapies.

VEGF is a secreted protein with powerful angiogenic properties that promotes angiogenesis in tumors, chronic inflammation, and wound healing. The mammalian VEGF family consists of five glycoproteins: VEGFA, VEGFB, VEGFC, VEGFD, and placenta growth factor^10,11^. Among the mammalian VEGF family members, VEGFA is a critical mediator of angiogenesis and is overexpressed in a variety of cancers^12–14^. VEGFA is a soluble predictive biomarker in circulating blood, and its significance during anti-angiogenic therapy has been investigated in various cancers, including gastric, ovarian, and colorectal^15^. Assessing the VEGFA level in the blood of ovarian cancer and metastatic colorectal cancer patients is a simple and cost-effective way to monitor the effects of anti-angiogenic therapies during treatment. High VEGFA levels in blood correlate with advanced tumor stage and poor survival outcomes in ovarian cancer patients^16–18^ and the progression-free and overall survival of metastatic colorectal cancer patients^19^. Thus, VEGFA is a useful biomarker for screening patient responsiveness to anti-angiogenic therapies.

In this study, we used a yeast two-hybrid screening analysis to identify a novel VEGFA-interacting protein, and we provide evidence identifying calpain-6, a unique nonconventional calpain without proteolytic activity, as a novel VEGFA-interaction protein. Using gene overexpression and gene silencing approaches, we demonstrate the significance of the VEGFA–calpain-6 interaction in angiogenesis promotion: calpain-6 enhances the secretion of VEGF. To the best of our knowledge, this is first report to identify calpain-6 as a VEGFA-interacting positive regulator of angiogenesis. Identification of this novel VEGFA-interaction protein could uncover mechanisms that underlie intrinsic or acquired drug resistance and provide insights for a novel anti-angiogenic strategy.

## Results

### Identification of calpain-6 as a novel VEGFA-interaction protein

**Figure 1:**
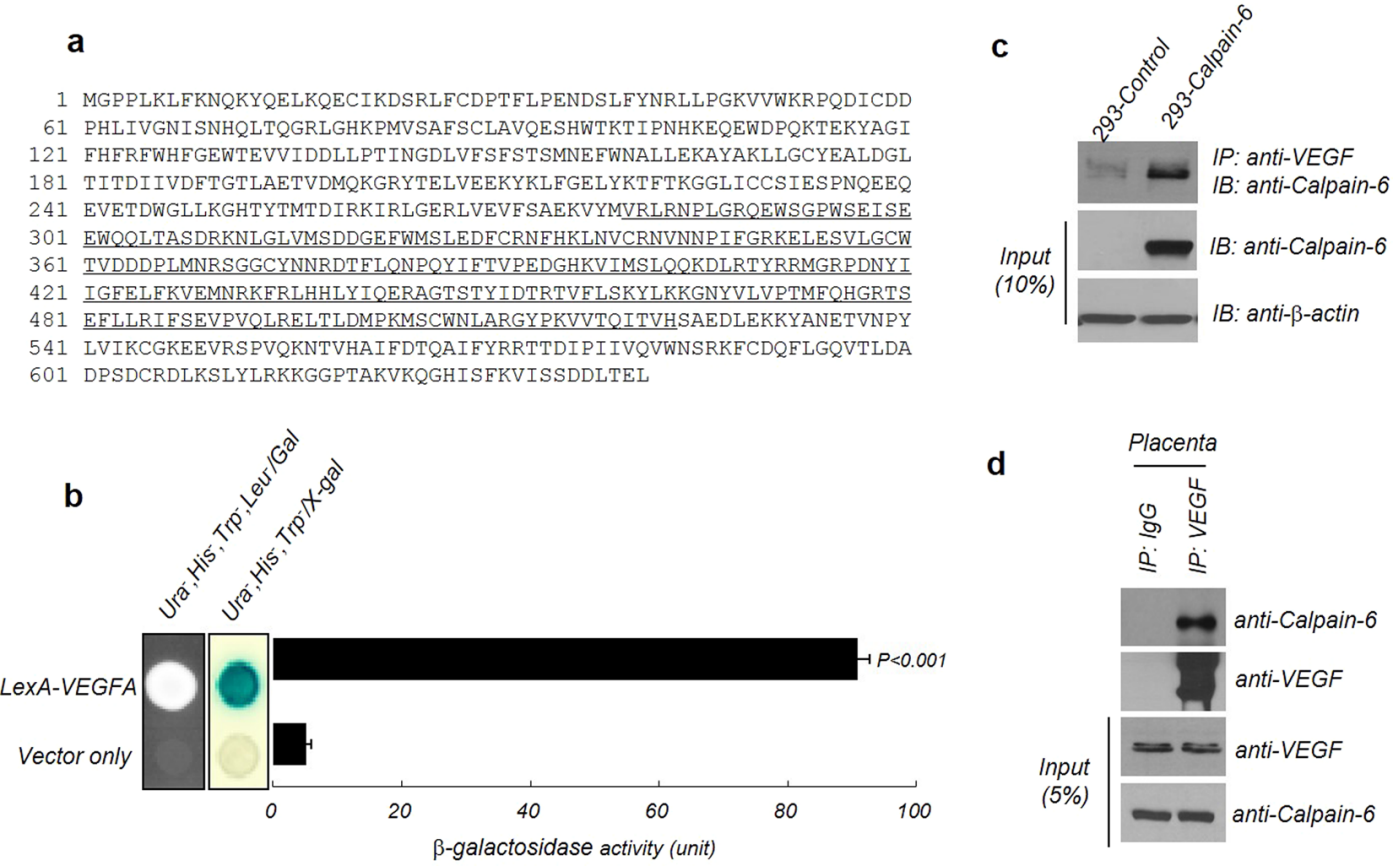
VEGFA interacts with calpain-6. (**a**) The amino acid sequence of calpain-6 depicted using single letter abbreviations. The underlined amino acid sequence (279–523) is the translated calpain-6 protein, isolated from the yeast two-hybrid screening analysis. (**b**) Identification of calpain-6 as a novel VEGFA-interaction protein through yeast two-hybrid screening. To test the interactions between VEGFA and calpain-6, both VEGFA and calpain-6 were expressed as pGilda and pJG4–5 fusion proteins in yeast, and β-galactosidase lift assays were done in the presence of X-gal to assess the binding activity of the constructs. Positive interaction was revealed by cell growth for 3 days at 30 °C on leucine-depleted medium, as well as by the formation of blue colonies on medium containing X-gal. Their interaction was quantitated using the relative activity of β-galactosidase in ONPG assays. B-galactosidase activity was normalized to the value obtained with full-length VEGFA. (**c**) Co-immunoprecipitation assay for the VEGFA–calpain-6 interaction in HEK293 cells. HEK293 cells were transiently transfected with a calpain-6-overexpressing construct and pcDNA3.1 The Myc-HisA control vector was immunoprecipitated with anti-VEGF, followed by immunoblotting with anti-calpain-6. β-actin was used as an equal loading control. (**d**) Endogenous calpain-6 and VEGFA interaction in human placental tissue. Placental tissue lysates were prepared in tissue lysate buffer and then subjected to immunoprecipitation with anti-VEGFA antibody, followed by immunoblot analysis with anti-calpain-6.

To identify a novel protein that interacts with VEGFA, we used the yeast two-hybrid screening assay. The results identified calpain-6 as a VEGFA-interaction protein. The underlined amino acid sequence (279–523) is the translated calpain-6 protein isolated from the yeast two-hybrid screening analysis (Fig. 1a). The interaction between VEGFA and calpain-6 was verified by the yeast growth assay and β-galactosidase assay *in vivo* (Fig. 1b). To examine the interaction between VEGFA and calpain-6 in human cells, immunoprecipitation assays were performed in Human fetal kidney epithelial cells (HEK293 cells) transfected with a calpain-6-expressing vector or a control vector. Immunoblots confirmed both VEGFA and calpain-6 in the calpain-6 immunoprecipitants from the calpain-6-expressing HEK293 cells, indicating an interaction between VEGFA and calpain-6 in HEK293 cells (Fig. 1c). To further confirm that they interact endogenously, human placental tissue, whose reported protein expression data indicated high calpain-6 expression, was used for an immunoprecipitation assay. A strong interaction between VEGFA and calpain-6 was detected in the human placental tissue lysates (Fig. 1d). These results suggest that VEGFA and calpain-6 interact with each other in yeast, cultured human cells, and human placental tissue.

### Calpain-6 enhances VEGF secretion in HEK293 cells

**Figure 2:**
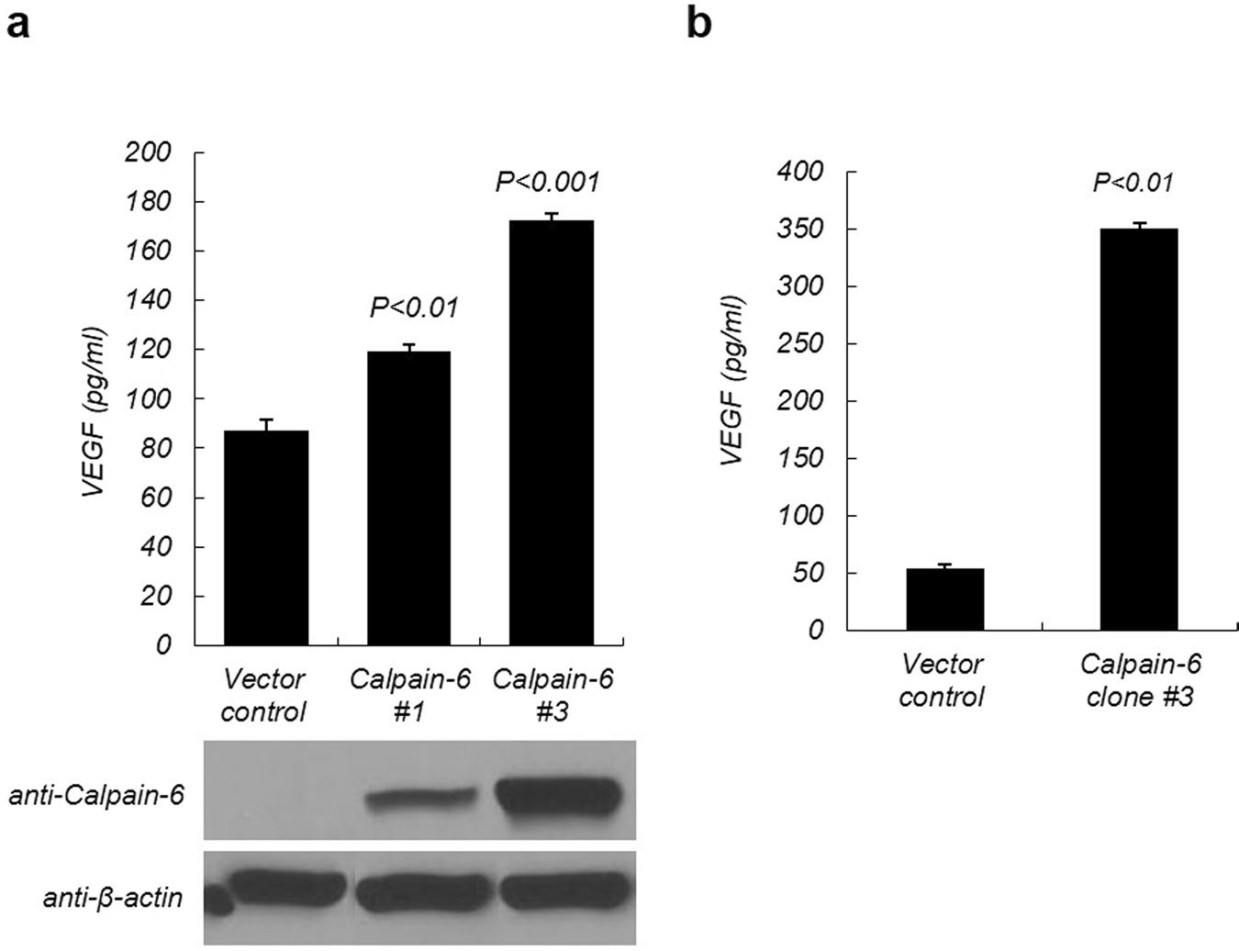
VEGFA secretion is enhanced by calpain-6 expression. (**a**) Calpain-6 increased VEGF secretion in a dose-dependent manner. HEK 293 cells were transiently transfected with calpain-6-overexpressing and control vector and then calpain-6 protein levels were confirmed by immunoblot analysis and ELISA in the cell lysates. (**b**) Establishment of vector stable control vector and calpain-6 overexpressing HEK293 cells. Calpain-6 overexpressing and control vector only expressing HEK293 cells were further selected in the presence of G418-containing culture medium for 3 weeks and then monoclonal vector control and calpain-6 expressing monoclonal cells were expanded. Conditioned medium (CM) was harvested and then processed for VEGF quantitation using an ELISA analysis. Data represent the mean ± SD of three independent experiments.

To explore the role of calpain-6 in human cells, we wanted to generate HEK293 cells that stably expressed either human calpain-6 or a control vector. The control vector- and calpain-6-expressing constructs were transiently transfected into HEK293 cells and then the expression of calpain-6 protein and VEGF secreted in serum-free conditioned medium (CM) were assessed by immunoblot and ELISA analysis, respectively. Secretion of VEGF protein in the transfected HEK293 cells was higher in the calpain-6-overexpressing cells than in the control cells in a dose-dependent manner (Fig. 2a). To generate vector-control and calpain-6-overexpressing stable cells, we further selected the transfectants in the presence of G418 and obtained vector control and calpain-6 expressing stable transfectants showing the highest calpain-6 expression. Clonally purified calpain-6-overexpressing HEK293 cells (clone #3) secreted 7-fold more VEGF in their CM than the vector control cells (Fig. 2b) and were used throughout this study.

### Enhancement of VEGF-induced angiogenesis by calpain-6 overexpression

**Figure 3:**
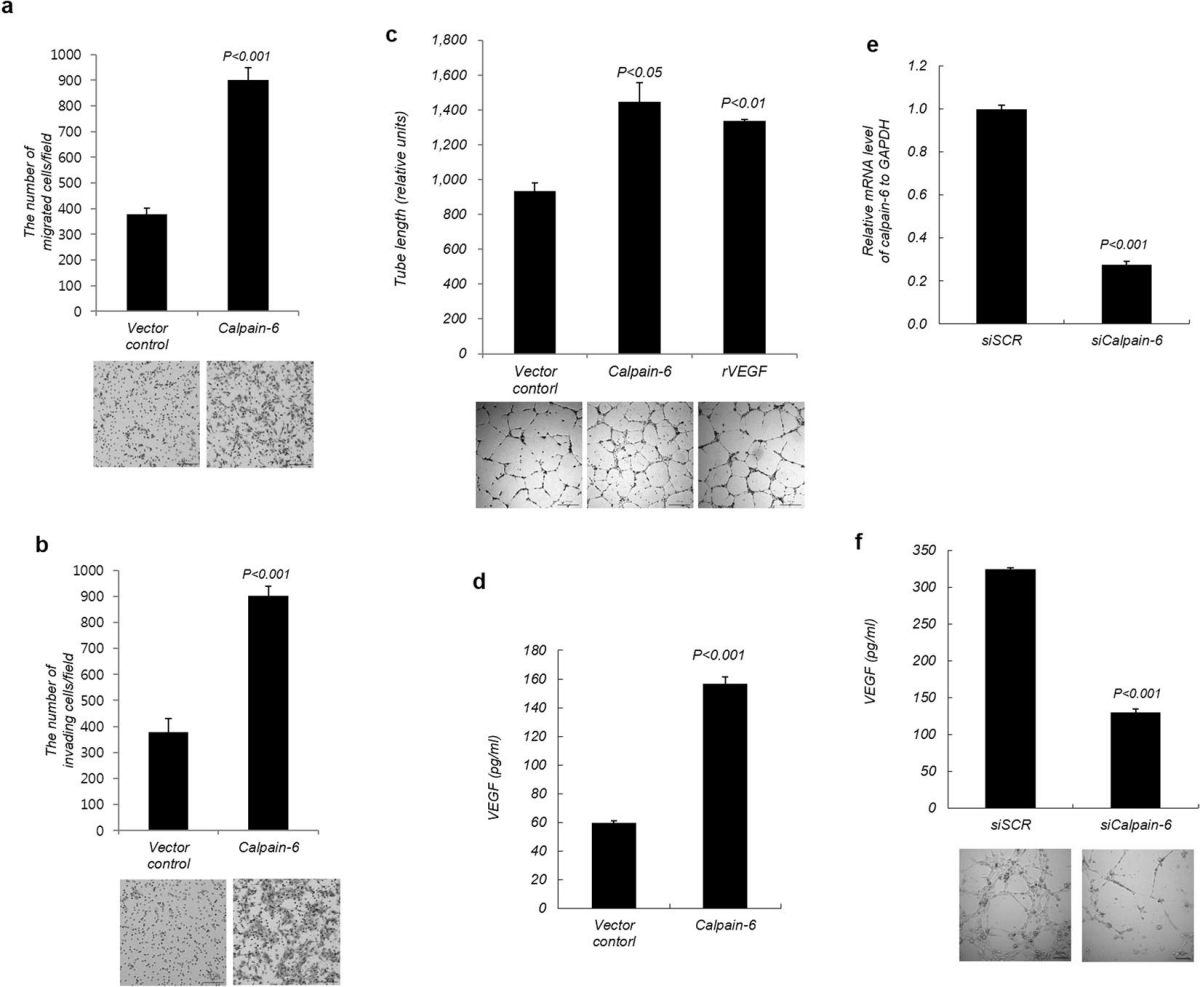
Calpain-6 promotes angiogenesis *in vitro*. (**a,b**) Secreted VEGF by calpain-6 increased HUVEC migration and invasion. HUVECs seeded on Transwells for the migration assays (**a**) or on Matrigel-coated Transwells for the invasion assays (**b**) were co-cultured with stable calpain-6-overexpressing or control vector HEK293 cells for 3 h. The number of migrated or invaded cells was photographed under a light microscope, and mean values were determined. (**c)** Calpain-6 overexpression in HUVECs promotes tube formation *in vitro*. Tube formation assays were performed with HUVECs transfected with control vector or calpain-6 expressing vector. As a positive control, HUVECs were incubated with recombinant human VEGF (10 ng/mL). The formation of tubular structures was captured by an inverted light microscope, and tube lengths were quantified by Inform. (**d**) Calpain-6 expression increased VEGF secretion in HUVECs transected with a calpain-6 expressing vector. VEGF protein in the conditioned medium of HUVECs transfected with control vector or calpain-6 expressing vector was analyzed by ELISA and the results were expressed as mean VEGF produced in the conditioned medium. Data represent the mean ± SD from three independent experiments. (**e**) Silencing of calpain-6 expression by siRNA. Calpain-6 stable HEK 293 cells were transfected with scrambled (SCR) and calpain-6 siRNA, respectively, and then analyzed the relative calpain-6 mRNA expression by real-time PCR. The results were expressed as the mean values. (**f**) Silencing of calpain-6 by siRNA decreased the VEGF secretion in HEK293 cells and leads to reduction in tube formation in HUVECs. The quantitation of VEGF secreted in the CM of stable HEK293-calpain-6 cells by calpain-6 siRNA was analyzed by ELISA and the results are expressed as mean VEGF produced in the conditioned medium. Data represent the mean ± SD of three independent experiments.

To explore the function of calpain-6 overexpression in angiogenesis, stable calpain-6-overexpressing HEK293 cells (purified clone#3) were used as a source of calpain-6 for *in vitro* angiogenesis assays in human vascular endothelial cells (HUVECs). We tested the effect of calpain-6 on HUVEC growth using a real-time cell electronic sensor system. Calpain-6 overexpression in HEK293 cells increased HEK 293 cell growth but CM derived from calpain-6 overexpressing HEK293 cells revealed no significant difference in HUVEC growth compared with vector control cells (data not shown). Based on our results indicating that calpain-6 overexpression increased the secretion of VEGF (Fig. 2), we expected calpain-6 to positively regulate angiogenesis. We next investigated whether the enhanced secretion of VEGF caused by calpain-6 would increase the migration and invasion of HUVECs, and we found that it did (Fig. 3a,b). These effects were transient, lasting for 3 h and no longer effective after 6 h (data not shown). To gain an insight into the effect of calpain-6 on angiogenesis, HUVEC cells were transiently transfected with a calpain-6-expressing construct and then examined in tube formation assays. Transfecting a calpain-6 construct into HUVECs markedly increased VEGF secretion and tube formation compared with the vector control (Fig. 3c,d), and the level of tube formation enhancement exceeded that of recombinant VEGF treatment. Together, these results indicate that calpain-6 enhanced the angiogenic properties of HUVECs. To further confirm whether calpain-6 is responsible for angiogenesis regulation, we knocked down calpain-6 in stable HEK293 calpain-6 overexpressing cells using an siRNA silencing approach. Silencing of calpain-6 by calpain-6-specific siRNA transfection efficiently suppresses exogenously expressed calpain-6 in HEK293-calpain-6 cells. The mRNA level of calpain-6 in siCalpain-6-treated HEK293 cells was significantly decreased compared with calpain-6 stable cells treated with scrambled siRNA (~70% knockdown of calpain-6 mRNA expression, Fig. 3e). To further investigate the effect of the loss of function by calpain-6-specific siRNA, tube formation assay in HUVEC cells was performed with CM collected from the control scrambled siRNA and calpain-6 specific siRNA transfected HEK293-calpain-6 cells. Silencing of calpain-6 in HEK293-calpain-6 cells significantly decreased the level of VEGF protein secreted in the CM and subsequently reduced the tube formation of HUVECs (Fig. 3f). These results suggested that calpain-6 regulated VEGF secretion and subsequently enhanced angiogenesis of HUVECs *in vitro*.

### Deletion mapping analysis of calpain-6 and VEGFA proteins

**Figure 4:**
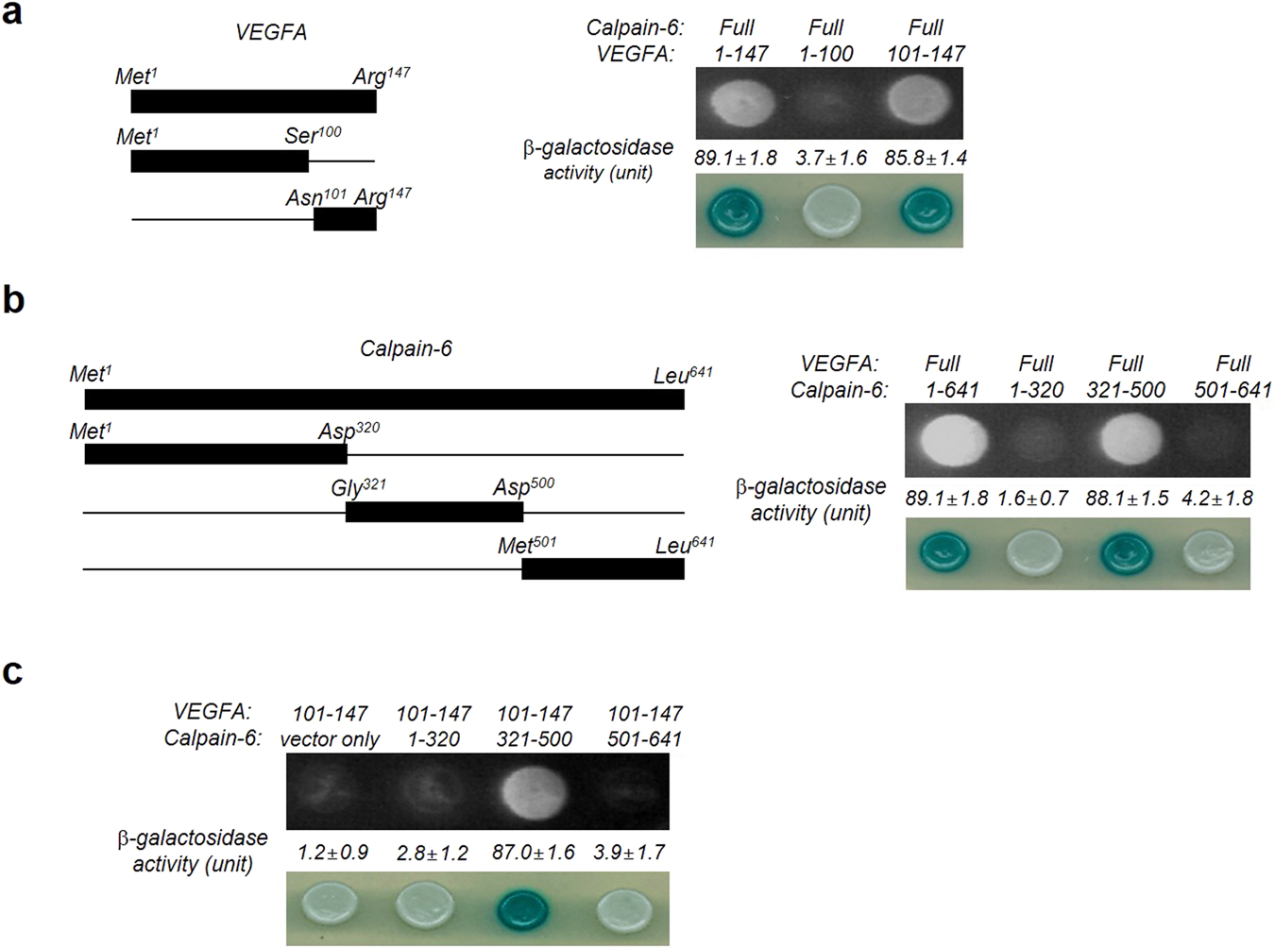
Domain mapping analysis of the VEGFA–calpain-6 interaction using the yeast two-hybrid assay. (**a**) The *left* panel is a schematic diagram showing the respective cDNA constructs for the VEGFA full-length and deletion mutants constructed by standard cloning and then verified by DNA sequencing. In the *right* panel, yeast transformants were assayed for their ability to grow on medium lacking leucine (upper panel) and for galactosidase expression through the formation of a blue colony on the plate containing X-gal (bottom panel). The values of β-galactosidase activity (unit), examined by adding *o*-nitrophenyl β-D-galactopyranoside (ONPG) agents, are indicated below their corresponding lanes. The data represent three independent experiments and are shown as means ± SD. (**b**) Identification of the interacting domains between calpain-6 and VEGFA through a yeast two-hybrid analysis. The *left* panel displays a schematic representation of the cDNA constructs for the full-length and truncated mutant calpain-6 fusion proteins. The *right* panel shows the results of the protein–protein interactions assessed in the yeast two-hybrid assay. The yeast transformants were assayed for their ability to grow on medium lacking leucine (upper panel) and for galactosidase expression through the formation of a blue colony on a plate containing X-gal (bottom panel). (**c**) Biological interactions between the cDNA constructs for VEGFA (Asn101–Arg147) and three calpain-6 deletion proteins (Met^1^–Asp^320^, Gly^321^–Asp^500^, Met^501^–Leu^641^), as determined by the yeast two-hybrid analysis. The C-terminal of VEGFA interacted with the domain comprising the amino acid region 321–500 in calpain-6.

To identify the specific region responsible for the interaction between VEGFA and calpain-6, we generated three VEGFA-expressing constructs: full-length VEGFA (Met^1^–Arg^147^), N-terminal VEGFA (Met^1^–Ser^100^), and C-terminal VEGFA (Asn^101^–Arg^147^) (Fig. 4a, *left* panel). We analyzed the interactions between the full length calpain-6 and the VEGFA truncation mutants using yeast two-hybrid assays. EGY48 yeast cells containing full-length VEGFA (Met^1^–Arg^147^) and those containing the C-terminal VEGFA (Asn^101^–Arg^147^) grew on the Ura, His, Trp, and Leu deficient plates, but yeast cells containing the N-terminal VEGFA (Met^1^–Ser^100^) failed to grow (Fig. 4a, *right* upper panel). We calculated the binding activity of these constructs by determining the relative expression levels of β-galactosidase. As indicated in the *right* lower panel in Fig. 4a, the β-galactosidase assay results confirm that the N-terminal VEGFA (Met^1^–Ser^100^) failed to bind calpain-6. In contrast, the C-terminus of VEGFA (residues 101–147) did interact with calpain-6 (Fig. 4a, *right* lower panel). Next, to localize the VEGFA binding domain on calpain-6 (Fig. 4b, *left* panel), full-length human VEGFA and full-length and three deletion mutants of human calpain-6 (Met^1^–Asp^320^, Gly^321^–Asp^500^, Met^501^–Leu^641^) were transformed in EGY48 yeast cells. Both the full length calpain-6 and the truncated form containing residues 321–500 interacted with the full length VEGFA (Fig. 4b, *right* upper panel). However, the calpain-6 deletion constructs containing the 1–320 and 501–641 residues, lacking domain III (321–500), lost the ability to bind VEGFA, suggesting the importance of domain III in the interaction with VEGFA (Fig. 4b, *right* panel). The efficiency of the interactions, as assessed by the relative expression levels of β-galactosidase between the pLexA-VEGFA fusion and calpain-6 deletion mutant, was comparable to that of the full-length constructs (Fig. 4b, *right* lower panel). Further analysis with the indicated deletion constructs of VEGFA and calpain-6 confirmed that the C-terminal domain of VEGFA was essential and sufficient for the interaction with calpain-6 (Fig. 4c). Only yeast cells containing the VEGFA C-terminal domain (Asn^101^–Arg^147^) and calpain-6 domain III (Gly^321^–Asp^500^) grew on the Ura, His, Trp, and Leu deficient plates, and the β-galactosidase analysis confirmed those results (Fig. 4c).

### Domain III of calpain-6 is necessary for calpain-6-mediated VEGF secretion

**Figure 5:**
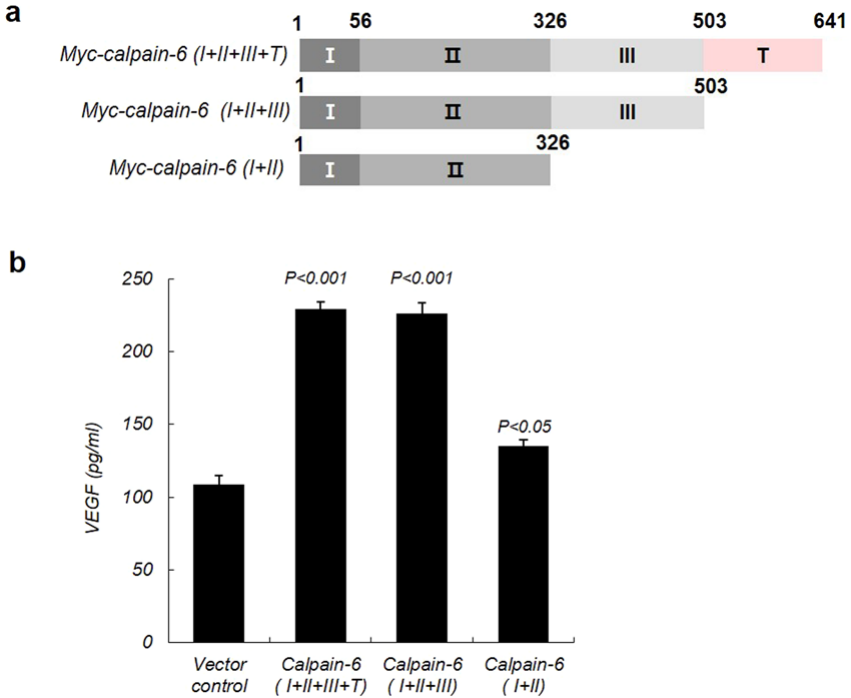
The VEGFA–calpain-6 interaction is necessary for calpain-6-mediated VEGF protein secretion. (**a**) Schematic diagram of the Myc-tagged full-length calpain-6 and deletion mutants, showing the deletions and domains I, II, III, and T. (**b**) Calpain-6 domain III is necessary for calpain-6-mediated VEGF secretion. The indicated full-length and mutant calpain-6 constructs were transiently transfected into HEK293 cells, and then the VEGF protein secreted into the conditioned culture medium was assayed by ELISA. The results are expressed as the mean VEGF produced in the conditioned medium ± SD. *P* < 0.001 or *P* < 0.05 compared with the control vector.

To further explore whether the calpain-6 interaction with VEGFA is functionally critical for calpain-6-mediated VEGF secretion, we assessed VEGF secretion using ELISA. Full-length calpain-6 and the truncation mutant constructs were separately, transiently expressed in HEK293 cells, and the conditioned culture media were collected to determine VEGF secretion (Fig. 5a). VEGF secretion was increased 2-fold upon the expression of calpain-6 domain III (Fig. 5b). In contrast, calpain-6 without domain III failed to increase VEGF secretion. Thus, domain III of calpain-6 is important to the increased secretion of VEGF through a protein–protein interaction. Deleting domain T of calpain-6 did not significantly change the secretion of VEGF compared with full-length calpain-6. Our results thus suggest that domain III of calpain-6 is important to calpain-6-mediated VEGF secretion.

## Discussion

VEGF is a secreted protein that powerfully promotes angiogenesis in tumors, chronic inflammation, and wound healing. The mammalian VEGF family consists of five glycoproteins: VEGFA, VEGFB, VEGFC, VEGFD, and placenta growth factor. Among them, tumor angiogenesis primarily relies on VEGFA-driven responses, producing dysfunctional vasculature in tumors^20,21^. Therefore, understanding the mechanisms of VEGFA is critical to establishing better strategies for cancer treatment. Although several anti-angiogenic therapies have been established, they have failed to improve survival outcomes in advanced cancers^10,11^, and their use has been associated with significant nephrotoxicity^9,22^, hypertension, and proteinuria because of a decrease in the production of vasoactive intermediates that occurs when VEGFA activity is inhibited, which in turn leads to vasoconstriction^23^. The existing strategies for treating angiogenesis thus have limited efficacy, and the best way to treat angiogenesis and endothelial dysfunction in cancer remains a matter of substantial debate^24,25^. Due to the significance of VEGF in both normal and pathological conditions, the identification of a novel VEGF-interacting protein might be useful in developing new treatment strategies. The identification and exploration of new targets for therapeutic intervention in angiogenesis inhibition is critical to improving the therapeutic efficiency of cancer treatments, especially for metastatic cancer.

In this study, we set out to identify a novel target for angiogenesis regulation and performed a yeast two-hybrid screening assay to identify a novel VEGFA-interaction protein. We successfully identified calpain-6 as a novel VEGFA-interaction protein and mapped the interacting domains in both proteins. We confirmed a strong interaction between VEGFA and calpain-6 in human placental tissue lysates (Fig. 1). Our yeast two-hybrid assays and immunoprecipitation assays revealed that domain III of the calpain-6 protein binds to VEGFA and that their interaction is functionally important to the enhancement of VEGF secretion (Figs 4 and 5).

Accumulating research suggests that conventional calpains play a central role in tumor migration and invasion through several key processes, including focal adhesion dynamics, cytoskeletal remodeling, epithelial-to-mesenchymal transition, and apoptosis^26^. Conventional calpains induce limited cleavage or the functional modulation of various substrates that serve as metastatic mediators upon activation. In calpain-1 and calpain-2, domain III constitutes a pair of four-stranded antiparallel β-sheets^27,28^ and seems to be involved in regulating Ca^2+^ sensitivity^27,29^ and Ca^2+^-dependent translocation to the cell membrane^30^. A BLAST search revealed that domain III of calpain-6 (amino acids 327 to 503) and calpain-2 share only 28% amino acid identity, suggesting limited conservation of the domain structure. In addition, domain III of calpain-6 lacks sequences that correspond to the conserved acidic loop that forms the interdomain salt bridges important for Ca^2+^ sensitivity^31^. Thus, domain III of calpain-6 could be unusual in structure and function in the calpain family, and its interaction with VEGFA might be unique. Our study provides the first evidence that the non-proteolytic calpain-6 is a novel VEGFA-interaction protein and demonstrates its critical function in angiogenesis. Previous studies showed that domain II of calpain-6 functions as a potential regulator of Rac1-mediated lamellipodial formation and enhances cell motility by interacting directly with the Rho guanine nucleotide exchange factor GEF-H1^32^. Calpain-6 exerts microtubule binding and stabilizing activity and participates in crosstalk between microtubules and actin filaments, which is crucial for various cellular functions, including cell migration, spreading, and cytokinesis^31,33^. Furthermore, calpain-6 promotes the microtubular association of GEF-H1 and prevents GEF-H1 from activating Rac1-mediated microtubule–actin crosstalk. These previous findings suggest that non-proteolytic calpain-6 functions as a structural regulator and chaperone for other molecules^32^.

Here, we propose a protein–protein interaction between VEGFA and calpain-6 as a novel mechanism of the angiogenesis-promoting function of calpain-6, an unconventional calpain that lacks protease activity because of the substitution of a cysteine residue for lysine in its active core^34,35^. Because angiogenesis requires protease activity and VEGF-induced cytoskeletal reorganization plays a crucial role in angiogenic processes, a role for calpain-2 in VEGF-induced angiogenesis was anticipated as previously reported^36^. However, the interaction between calpain-6 and VEGFA had not previously been investigated. We speculated that, instead of cleaving protein substrates (including tumor mediators), calpain-6 would interact with VEGFA to promote VEGF secretion, which would enhance angiogenesis. Herein, we have presented two major findings. First, we found that overexpression of calpain-6 can enhance angiogenesis by promoting VEGF secretion. Second, our domain mapping assays clearly show that domain III of calpain-6 is essential for both the physical interaction with VEGFA and the induction of VEGF secretion (Figs 4 and 5). Taken together, our findings suggest that calpain-6 binds to VEGFA in the cytoplasm, through domain III of calpain-6 and the C-terminus of VEGFA, and thereby promotes VEGF secretion. This enhanced secretion of VEGF can subsequently promote angiogenesis. In contrast, the inhibition of calpain-6 expression in the siRNA experiment abrogated calpain-6-induced angiogenesis *in vitro*. Future studies should examine the mechanisms by which the interaction between calpain-6 and VEGFA leads to an increase in VEGF secretion to promote angiogenesis and also whether other factors whose expression may be increased by the overexpression of calpain-6.

The role of non-proteolytic calpain-6 has been investigated, and previous studies have shown that calpain-6 regulates microtubule network stability during cytokinesis^31^ and exerts its inhibiting function in skeletal muscle cell differentiation during development and regeneration in mice^33^. Tonami *et al*. found that the deletion of *calpain-6* in mice promoted the development of embryonic skeletal muscle^31^, and they showed that calpain-6 was induced during the regeneration of skeletal muscles in adult mice after cardiotoxin-induced degeneration. Furthermore, *calpain-6* deficiency accelerated skeletal-muscle regeneration in mice. Interestingly, recent findings using calpain-6 regulatory sequence–based reporter assays identified *calpain-6* as a target gene for a stemness pathway in embryonic stem and cancer cells^33^. Researchers found that calpain-6-expressing cells were tumor-initiating and conferred the property of chemoresistance^37–41^, suggesting an association between calpain-6 and the malignant characteristics of sarcoma cells.

Clinical data have shown that 20% to 30% of all cancer patients experience muscle atrophy caused by cancer-associated cachexia^42^. Interestingly, previous reports showed that calpain-6 exerts a suppressor function on skeletal muscle differentiation in mice^33^ and plays a pivotal role in muscle regeneration. Those results suggest calpain-6 as a therapeutic target for muscle dystrophy/atrophy and suggest that it could be a useful tool in tissue engineering. Therefore, targeting calpain-6 as a cancer therapy could provide two benefits: inhibition of angiogenesis and prevention of cancer-associated cachexia manifested as muscle atrophy.

The current understanding of the involvement of calpain-6 in tumorigenesis is somewhat complex. Calpain-6 was found to be aberrantly expressed in different tumors, and it exhibits both anti-apoptotic and pro-angiogenic functions. Calpain-6 is overexpressed in many types of tumors and induces apoptosis resistance, cell proliferation, and angiogenesis in tumors^37–41^. The association between elevated calpain-6 expression and the inhibition of apoptosis and chemoresistance^37,39,43^ suggests its role in the malignant progression of primary tumors. Our previous study also showed that calpain-6 overexpression induced tumorigenesis by inhibiting cisplatin-mediated apoptosis and facilitating VEGF-mediated angiogenesis^44^. Our preliminary results with human ovarian tumor tissues by immunohistochemistry and immunoblot analysis suggested the possible correlation between VEGF expression and calpain-6 expression in that specific subtype of ovarian cancer but further investigation with a number of other specimens will be required to clarify this issue. In contrast, expression of calpain-6 was downregulated in head and neck squamous cell carcinoma (HNSCC), and decreased expression of calpain-6 was associated with tumorigenesis and poor prognosis in HNSCC, suggesting that calpain-6 could function as a tumor suppressor protein in HNSCC^45^. Therefore, the precise mechanisms underlying the various effects of calpain-6 in different human tumors remain to be elucidated and require further investigation.

In summary, we identified the non-proteolytic calpain-6 as a novel protein interacting with VEGFA, and we showed that calpain-6 promotes angiogenesis by increasing VEGF secretion. Our findings provide insights for the development of novel anti-angiogenic strategies in the treatment of many diseases.

## Methods

### Cell culture

HEK293 cells and HUVECs were purchased from the American Type Culture Collection (Minneapolis, MN, USA). HEK293 cells were cultured in Eagle’s minimum essential medium (EMEM) supplemented with 10% FBS and 1% penicillin/streptomycin (Invitrogen Corporation, Carlsbad, CA, USA). HUVECs were cultured in EGM^TM^-2 BulletKit^TM^ Medium (Lonza, Basel, Switzerland). Both cell lines were incubated under a 5% CO2 humidified atmosphere at 37 °C.

### Screening and interaction domain mapping through a yeast two-hybrid analysis

VEGFA was used as the bait, and a human cDNA library prepared from a human embryonic ovary was used as the prey. The EGY48 yeast strain and Matchmaker LexA Two-Hybrid system (Clontech, Palo Alto, CA, USA) were used to perform the yeast two-hybrid assay according to the manufacturer’s instructions. To generate the bait plasmid, the wild-type VEGFA gene was amplified by PCR and cloned into the pGilda vector using the *BamH I* and *Xho I* restriction sites. A human cDNA library prepared from a human embryonic ovary was used as the prey and inserted into a pB42AD prey vector. In addition, wild-type calpain-6 genes were amplified by PCR and cloned into the pB42AD prey vector between the *EcoR I* and *Xho I* restriction sites for the growth and ß-galactosidase assays. Truncated deletion mutants of VEGFA and calpain-6 were also generated in the prey and bait vectors, respectively. The primers used to clone all constructs were as follows: *VEGFA*(1–147), 5′-CGG GGA TTC GTA TGA ACT TTC TGC TGT CT-3′ (forward) and 5′-ATT CTC GAG TCA CCG CCT CGG CTT GTC ACA-3′ (reverse); *VEGFA*(1–100), 5′-CGG GGA TTC GTA TGA ACT TTC TGC TGT CT-3′ (forward) and 5′-ATT CTC GAG TTA GGA CTC AGT GGG CAC-3′ (reverse); VEGFA(101–147), 5′-CGG GGA TTC GTA ACA TCA CCA TGC AGA TT-3′ (forward) and 5′-ATT CTC GAG TCA CCG CCT CGG CTT GTC ACA-3′ (reverse); calpain-6(1–641), 5′-CGG GAA TTC ATG GGT CCT CTG AAG CTC-3′ (forward) and 5′-ATT CTC GAG TTA GAG CTC AGT GAG ATC ATC-3′ (reverse); calpain-6(1–320), 5′-CGG GAA TTC ATG GGT CCT CTG AAG CTC-3′ (forward) and 5′-CGG CTC GAG TCA ATC AGA CAT AAC AAG-3′ (reverse); calpain-6(321–500), 5′-CGG GAA TTC GGA GAG TTT TGG ATG AGC TTG-3′ (forward) and 5′-ATT CTC GAG TTA GTC CAG AGT CAG TTC CCT-3′ (reverse); calpain-6(501–641), 5′-ATT GAA TTC ATG CCC AAA ATG TCC TGC TGG-3′ (forward) and 5′-ATT CTC GAG TTA GAG CTC AGT GAG ATC ATC-3′ (reverse). All constructs were confirmed by sequencing. The bait and prey vectors were co-transformed in EGY48 yeast, and the transformants were grown for 3 days at 30 °C on plates in dropout media lacking uracil, histidine, and tryptophan. Positive colonies were confirmed using growth and β-galactosidase assays on plates lacking uracil, histidine, tryptophan, and leucine or containing X-gal, respectively. A positive interaction was defined as the ability to support cell growth on leucine-depleted media or the formation of blue colonies on the X-gal-containing media. The binding activity of the interaction was calculated using an *o*-nitrophenylβ-D-galactopyranoside (ONPG)-galactosidase analysis system as described^44^.

### Mammalian expression constructs

The wild-type or truncated versions of the human calpain-6 gene were PCR-amplified using specific primer sets: full length calapin-6 (1–641, I + II + III + T), 5′-CCG GGA ATT CAT GGG TCC TCT GAA GCT CT-3′ (forward) and 5′-CCG GCT CGA GGA GCT CAG TGA GAT CAT CGC TG-3′ (reverse); T-domain truncated calpain-6 (1–503, I + II + III), 5′-TGT CCA GAG TCA GT-3′ (forward) and 5′-CCG GCT CGA GTT TGG GCA-3′ (reverse); Domain III and T truncated *calpain-6* (1–326,I + II), 5′-AAA ACT CTC CAT CA-3′ (forward) and 5′-CCG GCT CGA GGC TCA TCC-3′ (reverse). Thermal cycling conditions used an initial step at 95 °C for 5 min followed by 35 cycles at 95 °C for 1 min, 55 °C for 1 min, 72 °C for 2 min, and then a final extension at 72 °C. Amplified calpain-6 sequences were then subcloned into the EcoR1-XhoI-digested mammalian expression vector pcDNA3.1 Myc-HisA (Invitrogen). The sequence of calpain-6 in the mammalian vector was confirmed by DNA sequencing.

### Transfection and generation of stable calpain-6-overexpressing HEK293 cells

HEK293 cells were seeded the day before transfection and cultured until they reached 60% confluence on the day of transfection. HEK293 cells were transiently transfected with a control vector or the calpain-6-expressing vector using Effectene (Qiagen, Hilden, Germany) according to the manufacturer’s instructions. Transfected cells were selected in medium containing G418 (500 μg/mL; Invitrogen), and G418-resistant discrete colonies were selected for further expansion in selection medium. Several clones of control vector and calpain-6-overexpressing HEK293 cells were selected with complete EMEM containing G418. The expression of calpain-6 was assessed by immunoblotting analysis and the clone#3 which showed the highest expression of calpain-6 was chosen and used throughout the study.

### Small interference RNA

Gene silencing in stable calpain-6-overexpressing HEK293 cells were achieved by transfection of siRNA directed against *CAPN6* (Bioneer, Daejeon, ROK) and non-targeting siRNA (siSCR) as a control. According to the manufacturer’s protocol, siRNA was added to OPTI-MEM media (Gibco) with Lipofectamin3000 (Invitrogen) and cells were incubated with complete medium. To avoid cytotoxicity, transfection medium was replaced with complete medium 4 h after siRNA transfection. For each experiment transfection efficiency was verified using real time quantitative PCR (RT-qPCR) at 48 h after siRNA transfection.

### Real-time PCR

Total RNA from *CAPN6* and siSCR siRNA-transfected stable calpain-6-overexpressing HEK293 cells were isolated with RNeasy Mini Kit (Qiagen) following the manufacturer’s specifications. The expression level of the caapain-6 was analysed by quantitative real-time polymerase chain reaction (qPCR) on QuantStudio6 PCR System (Applied Biosystems, CA, USA) using SYBR Green Master Mix (Qiagen) following manufacturers’ specifications. The relative expression of mRNA level was analysed by QuantStudio Real-Time PCR Software (Applied Biosystems) and the ΔΔCt method in order to obtain the relative *CAPN6* gene expression. *GAPDH* was used as endogenous control, and siSCR group as the reference control. To determine the inhibition of gene expression after siRNA transfection, the following primers were used: *CAPN6*, 5′-AGT ACC TGA AGA AGG GCA ACT-3′ (Forward) and 5′-CAG GTT CCA GCA GGA CAT TTT-3′ (Reverse); *GAPDH*, 5′-GCA CCA ACT GCT TAG C-3′(Forward) and 5′-GGC CAT CCA CAG TCT G-3′ (Reverse).

### Tissue and cell lysate preparation

Human placental tissue samples, which were obtained from Samsung Medical Center in 2000 and stored in an LN2 tank before IRB regulation, were homogenized in 1 ml of ice-cold homogenization buffer (20 mM HEPES [pH 7.4], 75 mM NaCl, 2.5 mM MgCl2, 0.2 mM EDTA, 0.05% Triton X-100, 20 mM β-glycerophosphate, 1 mM Na3VO4, 0.5 mM DTT, and 10 mMNaF) and protease inhibitor cocktail (Roche, Mannheim, Germany), as described previously^45^. Transfected cells were lysed by sonication in RIPA buffer (50 mM Tris-HCl [pH 8.0], 150 mM NaCl, 1% NP-40, 0.1% sodium dodecyl sulfate [SDS], and 10 mM sodium deoxycholate) in the presence of protease inhibitor cocktail (Roche). Lysates were centrifuged at 13,000 rpm for 10 min at 4 °C, and the protein concentration was measured using the Bradford assay (BioRad, Hercules, CA, USA). Tissue and cell lysates were immediately stored at −80 °C until further analysis.

### Immunoprecipitation and immunoblotting

Whole cell lysates from the cultured HEK293 cells and normal placental tissue (total 1 mg of protein) were incubated with VEGF antibody (Santa Cruz Biotechnology, Santa Cruz, CA, USA) overnight at 4 °C. Following incubation, immunocomplexes were immunoprecipitated using protein A/G agarose beads (Santa Cruz Biotechnology) for 2 h at 4 °C with gentle rotation. The beads were washed three times with lysis buffer and boiled in 50 μl of 1X SDS sample buffer for 5 min at 95 °C. After centrifugation, the precipitated proteins were separated by SDS-PAGE and electrophoretically transferred onto an enhanced chemiluminescence (ECL) nitrocellulose membrane (GE Healthcare, London, UK). Western blot analysis was performed with the indicated primary antibodies followed by appropriate horseradish peroxidase–conjugated secondary antibodies using an ECL system (GE Healthcare, Buckinghamshire, UK), as described previously^45^. Equal protein loading was confirmed by Ponceau S staining and sequential incubation of the membrane with anti-β-actin (Sigma-Aldrich) antibody.

### Analysis of secreted VEGF using an enzyme-linked immunosorbent assay (ELISA)

The amount of VEGF in the conditioned medium taken from transiently and stably transfected HEK293 cells was determined using the human VEGF Quantikine ELISA kit (DVE00, R&D Systems, Minneapolis, MN, USA) according to the manufacturer’s instructions.

### Migration and invasion assays

Migration and invasion were assayed using Transwells (8-μm pore size, Costar, Cambridge, MA). For the migration assay, the outer surface of the filter was coated with 10 mg of gelatin. Stable control vector or calpain-6-expressing cells were seeded into the lower wells in M199 media containing 1% FBS and allowed to attach and spread during a 24 h incubation. Then HUVECs were seeded into each of the upper wells at a final concentration of 1 × 10^5^/ml. The cells were incubated for the indicated times. Cells were fixed and stained with hematoxylin and eosin. Non-migrating cells on the upper surface of the filter were removed by wiping with a cotton swab. The cells that migrated to the lower side of the filter were photographed by light microscopy and counted using the ImageJ program (NIH, Bethesda, MD, USA). The mean values of four fields were determined as described^45^. For the invasion assay, the outer and inner surfaces of the filter were coated with 1 mg/ml of collagen and 0.5 mg/ml of Matrigel (BD Biosciences), respectively. The cell seeding condition and quantification methods were the same as in the migration assays. Independent experiments were repeated three times, and the data shown are the mean ± standard deviation (SD) of triplicate samples.

### *In vitro* tube formation assay

Growth factor–reduced Matrigel (200 μl of a 10 mg/ml stock) was added to a 24-well plate and polymerized for 30 min at 37 °C. HUVECs (1.5 × 10^5^ cells) transiently transfected with control vector or calpain-6-expressing vector were plated onto the surface of the Matrigel. Recombinant VEGF (293-VE, R&D Systems)-treated HUVECs were used as the positive control. The cells were incubated for 16 h in M199 medium containing 1% FBS. The formation of tubular structures was detected using an inverted microscope at 20X magnification. Tube lengths were quantified using the Inform (PerkinElmer, Waltham, MA, USA) analysis program. The data shown are the mean ± SD of triplicate samples, and independent experiments were repeated three times.

### Statistical analysis

All experiments were independently performed three times. The data presented are the mean ± SD. An unpaired Student’s t-test was used to assess statistical significance. Statistical significance was defined as *p* < 0.05, *p* < 0.01 and *p* < 0.001.
